# Silicosis and silicotuberculosis in India

**DOI:** 10.2471/BLT.15.163550

**Published:** 2016-08-31

**Authors:** Nandini Sharma, Debashish Kundu, Sunita Dhaked, Anand Das

**Affiliations:** a Department of Community Medicine, Maulana Azad Medical College, New Delhi, India.; b RNTCP Technical Support Network, Revised National Tuberculosis Programme, D-776, First Floor, C R Park, New Delhi, 110019, India.; c The International Union Against Tuberculosis and Lung Disease (The Union), South East Asia Regional Office, New Delhi, India.

Silicosis is a progressive interstitial lung disease, characterized by shortness of breath, cough, fever and bluish skin; it can present in three different forms: acute, accelerated and chronic.^1^ It is caused by the lung tissue reaction to the inhalation of silica and occurs most commonly as an occupational disease of people working in the quarrying, manufacturing and building construction industries. Exposure to large amounts of free silica can go unnoticed because silica is odourless, non-irritant and does not cause any immediate health effects. As silicosis is incurable, clinical management includes removing the worker from the industry and giving symptomatic treatment. Public health goals are to detect early cases through monitoring of currently and formerly exposed workers, to establish surveillance programmes, to slow progression and to reduce disability.^2^

In 1995, the World Health Organization and the International Labour Organization began a public awareness and prevention campaign to eliminate silicosis from the world by 2030.^3^Several countries – Brazil, Chile, China, Indonesia, Malaysia, Mexico, Poland, South Africa, Thailand, Turkey, Ukraine, the Bolivarian Republic of Venezuela and Viet Nam – have established national programmes for the elimination of silicosis.^3^ However, in many low- to middle-income countries, including India, silicosis continues to be an occupational health hazard.

India has a large mining industry, concentrated in the states of Chhattisgarh, Jharkhand, Orissa and West Bengal. In 1999, the Indian Council of Medical Research reported that around 3.0 million workers are at high risk of exposure to silica; of these, 1.7 million work in mining or quarrying activities, 0.6 million in the manufacture of non-metallic products (such as refractory products, structural clay, glass and mica) and 0.7 million in the metals industry.^4^ There are also around 5.3 million construction workers at risk of silica exposure.^4^ Due to variations in silica concentrations and duration of exposure in the work environment the reported prevalence of silicosis in India ranges widely – from 3.5% among 1977 workers in an ordnance factory to 54.6% in 593 workers in the slate-pencil industry.^5^^,^^6^ The main challenge of eliminating silicosis in India is in the informal, unregulated sectors of industry which do not fall under the control of statutory tools such as the Factory Act of India (1948).^7^ This Act mandates a well ventilated working environment, provisions for protection from dust, reduction of overcrowding and provision of basic occupational health care. Silicosis-affected workers in the informal sector are not entitled to statutory protection, which would remove them from the hazardous environment, or to compensation, which would enable them to leave work. Continued exposure makes it difficult for physicians to manage the disease. Furthermore, most primary-care physicians in India are not trained to manage occupational health diseases.

Among the clinical complications of silicosis is tuberculosis (called silicotuberculosis), a disease which is still a major public health concern in low- and middle-income countries.^8^ Chronic exposure to silica increases workers’ risk of tuberculosis infection and aggravates pre-existing pulmonary tuberculosis.^9^^–^^11^ Differential diagnosis is a challenge. Although treatable, tuberculosis in silicosis patients may go undiagnosed because cough, wheeze, expectoration, dyspnoea and vague chest pains are symptoms common to both diseases. Interpretation of the chest X-ray film of patients with silicosis is difficult due to the superimposition of silicotic nodules and tuberculous infiltrations. *Mycobacterium tuberculosis* bacilli may not be recovered from the sputum of silicotuberculosis patients because silicotic fibrosis prevents the discharge of tubercle bacilli into the sputum.^8^ Acid-fast bacilli, if cultured, are mainly non-tuberculous mycobacteria.

The National Human Rights Commission of India (NHRC) has directed the governments of the states and union territories of India to provide complete information about all measures taken to prevent and eliminate the problem of silicosis.^12^Acting on the NHRC’s recommendations, a comprehensive survey of organized and unorganized industries where silica exposure may occur was done in the state of Gujarat.^13^ Other measures included provision of free diagnostic and treatment facilities at primary-, secondary- and tertiary-level health facilities to workers exposed to silica, along with counselling of patients about how to avoid dust inhalation and prevent progression of the disease. Awareness is being raised through information materials printed in the local language. Silicosis health-care units have been established in silicosis-risk districts, where free chest X-ray and pulmonary function tests are done. Regular inspections are made of industries that use silica, with active involvement of nongovernmental organizations (NGOs) to ensure proper monitoring.^13^

Following the Gujarat experience, and based on insights gained from available knowledge, we recommend that a silicosis control programme be set up in India, focusing on five areas for action. First, an occupational health and dust survey along with clinical examination, chest radiography and pulmonary function tests of workers every six months should be made mandatory in potentially hazardous industries. Cost–effective engineering control measures to manage silica dust need to be developed and promoted. Silicosis is a notified disease under the Mines Act (1952) and the Factories Act (1948). It should also be made a notifiable disease under the Public Health Act (1875), so that reporting becomes mandatory. Awareness campaigns are needed to sensitize workers about their risk of silicosis, personal protective measures and early symptoms.

Second, the silicosis control programme should be integrated with the existing revised national tuberculosis control programme of India. District tuberculosis officers, in collaboration with the Ministry of Labour, must ensure documentation of workplaces and workers at risk from silica exposure, especially in the informal sector. Occupational history-taking must be mandatory to differentiate silicosis from pulmonary tuberculosis and hence avoid the risk of unnecessary anti-tubercular therapy for the former.^12^ In areas with silicosis-risk industries the sputum of suspected cases of tuberculosis should be cultured and given antibiotic susceptibility testing.

Third, training is needed for medical officers and other public health professionals, particularly those working in the national tuberculosis control programme, to ensure early diagnosis and detection of silicosis.

Fourth, we propose that the administration of compensation for silicosis-affected workers should be privatized to improve the efficiency of reimbursements. The national health insurance programme in India for households below the poverty line (called *Rashtriya Swasthiya Bima Yojna*, RSBY) uses an efficient, computerized network for tracking claims and for reimbursements through private health insurance or third-party administrators. The government may consider extending RSBY to poor workers who are at risk of contracting silicosis and to their families. Silicosis is a compensable injury under the Employees’ State Insurance Act (1948) and the Workmen’s Compensation Act (1923). If silicosis health boards were set up in every state of India they could carry out surveillance for silicosis cases and assessment of disability and loss of earnings resulting from silicosis so as to decide the level of compensation and rehabilitation.^12^ NGOs could be involved in active monitoring and implementation of the compensation programme. It would then be important to educate miners, particularly migrant workers, about the compensation system.^13^

Fifth, appropriate follow-up and counselling should be provided to patients affected by silicosis. This would be possible with integration of the silicosis control programme with the existing national tuberculosis control programme.

Despite many clinical and operational challenges in the management of silicosis and silicotuberculosis, there is an opportunity for the Government of India to formulate a comprehensive policy framework on prevention, treatment, rehabilitation, compensation and follow-up. Action needs to be taken on active case-finding for documenting the burden of silicosis and silicotuberculosis, as recommended by the NHRC.^12^ Guidelines are needed under the revised Indian national tuberculosis control programme towards management of those at risk of developing silicotuberculosis.
